# A unique case of a lymphoproliferative disorder affecting the skin and uterine cervix on a male transgender^☆^

**DOI:** 10.1016/j.abd.2023.02.006

**Published:** 2023-09-01

**Authors:** Jade Cury-Martins, Marcelo A. Giannotti, Denis Miyashiro, Juliana Pereira, José Antonio Sanches

**Affiliations:** a Department of Dermatology, Universidade de São Paulo, São Paulo, SP, Brazil; b Department of Pathology, Universidade de São Paulo, São Paulo, SP, Brazil; c Department of Hematology, Universidade de São Paulo, São Paulo, SP, Brazil

Dear Editor,

Extranodal lymphomas account for 25%-35% of all Non-Hodgkin's lymphomas (NHL). The most common site of involvement is the gastrointestinal tract, followed by the skin.^1^, ^2^

Primary cutaneous follicular center lymphoma (FCL) is the most common form of cutaneous B-cell lymphoma, accounting for 50%-60% of cases.^3^, ^4^ It affects mainly adults, and has an excellent prognosis and a specific five-year survival of more than 95%.^2^ Extracutaneous spread is extremely rare, but recurrences are frequent.^4^

Primary female genital system lymphoma (PFGSL) is extremely rare, accounting for 0.2%‒1.1% of all extranodal lymphomas, with an estimated number of 165 new cases annually in the USA.^1^ Secondary involvement by nodal lymphoma is more frequent. The most commonly affected site is the ovary, followed by the uterine cervix, and the most common histopathological subtype is diffuse large B-cell lymphoma, followed by follicular lymphoma.^1^, ^5^, ^6^

Regarding the risk factors, it is suggested that a possible chronic antigenic stimulus may have a causal role, but no factor has been well established yet. In cutaneous B-cell marginal zone lymphomas, a relationship with *Borrelia burgdorferi* has been suggested, but this association has not been confirmed.^7^ Regarding transgender individuals, the question of the role of hormonal stimulation in the development of neoplasms is important. For hormone-dependent neoplasms, such as breast cancer, this risk exists; for NHL there is a hypothesis of a hormonal role in some studies, but this relationship is suggested for female hormone replacement, but is not described for male hormones, as would be the case in transgender men, as in the case described below.^8^

The authors describe a 42-year-old male transgender patient, without comorbidities, who presented to the Dermatology Outpatient Clinic with an asymptomatic, slow-growing, infiltrated, erythematous tumor, measuring 3 cm in the left preauricular region (Fig. 1). Histopathology disclosed an infiltrate of small lymphocytes, with a “bottom-heavy” nodular growth pattern, and no epidermal alterations. The neoplastic cells were CD20+ and CD10 and MUM1 negative; with CD21 staining in the lymphoid follicles; BCL6+ both inside (more evident) and outside the follicles; BCL2+ in B and T cells; CD3 and CD5 revealed a background of reactive T cells but were negative in atypical cells (Fig. 2). Polymerase chain reaction (PCR) showed monoclonality for the IgH gene in the B cell population. A diagnosis of FCL was made and no evidence of extracutaneous disease was found after staging with CT scans of the chest, abdomen, pelvis and cervix, and a bone marrow biopsy. The patient was treated with intralesional corticosteroids with a complete response (Fig. 1B).

**Figure 1:**
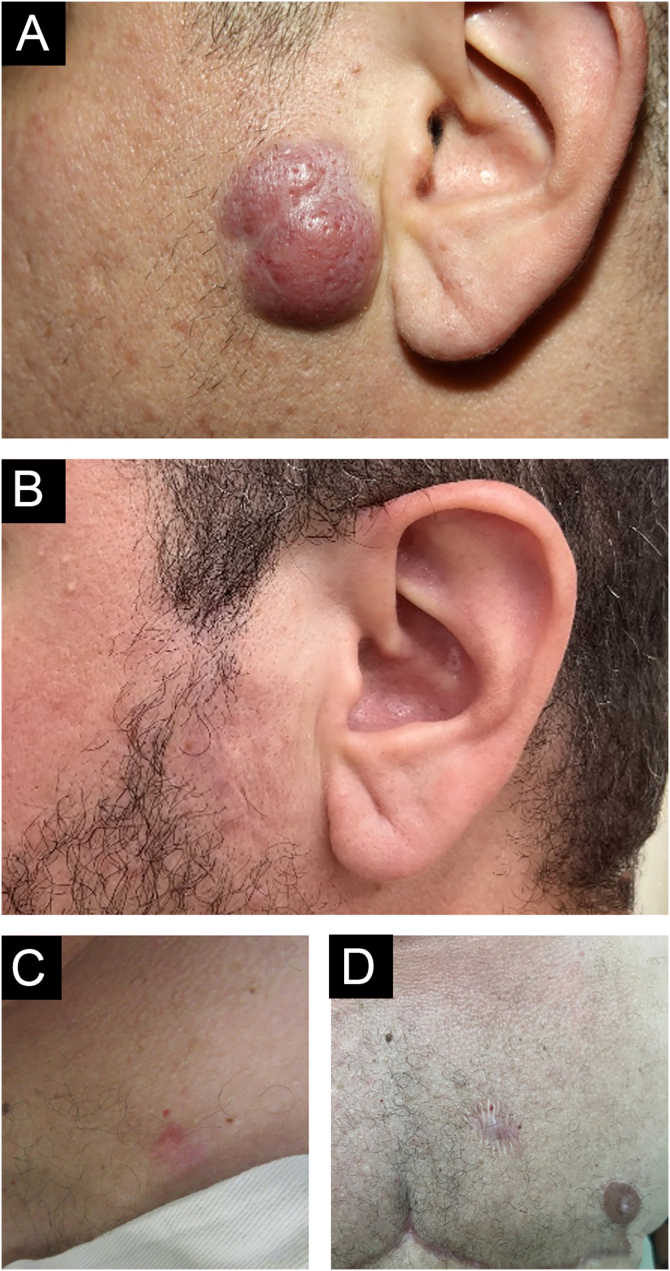
Clinical photo: (A) erythematous, infiltrated, asymptomatic, slow-growing tumor measuring 3 cm on the left preauricular region; (B) there was complete resolution after the use of topical and intralesional corticosteroids. (C) Erythematous nodule on the left breast and (D) resolution with scarring after excision

**Figure 2:**
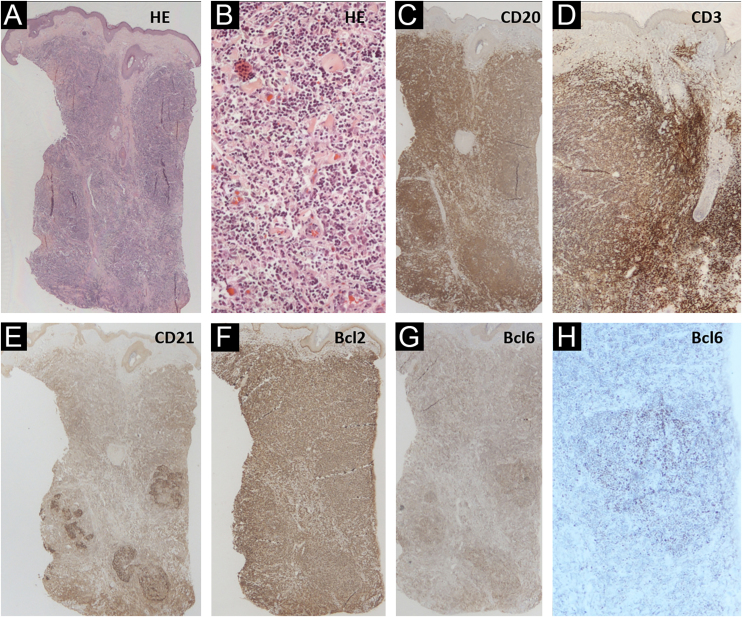
(A‒H) Skin histopathology: (Hematoxylin & eosin, ×10 and ×200), there is a “bottom-heavy” infiltrate of small lymphocytes, with a nodular growth pattern and no epidermal alterations; CD20 (×10) highlighting neoplastic B-cells; CD3 (×10) showing the associated rich T-cell infiltrate; CD21 showing areas with follicle outlines; Bcl2 positive (×10) in B and T cells; Bcl6 positive (×10 and ×40) inside and outside the germinal centers

Regarding gender identity, the patient started a hormonal transition (with testosterone) at the age of 40 years. Two years after the diagnosis of cutaneous lymphoma, the patient continued the transition by undergoing a total hystero-salpingo-oophorectomy. Histopathology of the uterine cervix disclosed lymphoid aggregates in a nodular, follicular pattern, with a phenotype similar to that of the skin lesion (CD20+, CD23 highlighting nodules, in a background rich in T CD3+ and CD5+ cells, weak BCL6+, with a strong similarity to the distribution of CD20+, CD10-, MUM-, Bcl2- cells in the germinal center, and resembling the distribution of CD3+ and KI67 cells, with lack of polarization inside the neoplastic follicles; Fig. 3). PCR analysis for immunoglobulin was inconclusive. The investigation of the t(14;18) translocation in paraffin-embedded tissue, both in the cervix and the skin of the face, was negative. The patient had no B symptoms and no signs of residual disease on PET-CT staging and a new bone marrow biopsy, thus, clinical follow-up without additional treatment was chosen.

**Figure 3:**
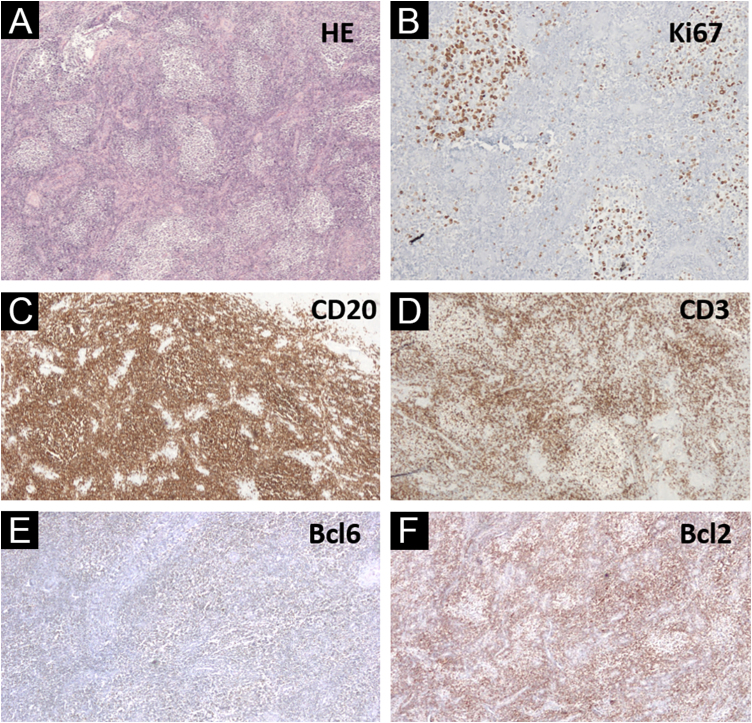
(A‒F) Uterine cervix histopathology (Hematoxylin & eosin, ×40), showing lymphoid aggregates in a nodular and follicular pattern; Ki67 (×200) with lack of polarization inside the neoplastic follicles; CD20 (×40) highlighting B cells outlining follicles; CD3 positive in cells around lymphoid follicles; weakly Bcl6 positive cells, but resembling CD20 positive cells; Bcl2 negative reaction (×40) in lymphoid follicles, with a distribution similar to CD3+ cells

Two years later, a new cutaneous nodule on the right thorax, with the same clinical aspect as the one on the face, was surgically excised, and presented histopathologic findings again compatible with the diagnosis of FCL (Figs. 1 C and D). The patient has been in complete remission for two years.

After several multidisciplinary discussions, no conclusion could be reached on the unification of the cutaneous and uterine findings. Initially, the diagnosis of FCL was made. However, extracutaneous spread of FCL is rare and, when present, is more frequent to local lymph nodes.^4^ No previous description of spread to the uterine cervix was found.

One possibility would be a primary nodal lymphoma secondarily affecting the skin and cervix. However, no signs of nodal involvement were found when staging with tomography and later through PET-CT, nor disease in the bone marrow biopsy, at the time of the diagnosis in the skin and cervix. Additionally, nodal lymphomas are usually Bcl2 positive in germinal centers.^9^

This led to a third proposition of two different primary extranodal follicular B-cell lymphomas: an FCL and a PFGSL. Also, for this association, no previous description was found.

Another possibility would be that the involvement of the uterine cervix represents a pseudolymphoma. Lymphoma-like lesions of the cervix were first described in 1985, and there are other rare reports.^5^, ^10^ Au et al. described three cases of uterine cervix B-cell lymphomas, but upon review concluded that two of the three were, in fact, pseudolymphomas. The reported cases usually regressed spontaneously when the biopsy was repeated. As possible diagnostic clues for pseudolymphoma, a more superficial distribution of the infiltrate is suggested, with heterogeneity of lymphoid cells and absence of masses or ulcerations, but this can also occur in indolent lymphomas. It can be suggested that the detection of the immunoglobulin gene rearrangement by PCR helps to differentiate these two diseases since the lymphoma is usually clonal while the pseudolymphoma, as a reactional and non-neoplastic process, is expected to be polyclonal. However, previous studies have shown that clonal rearrangement can also be detected in benign conditions, reported, for instance, in 50% of benign gastric lymphoid hyperplasia cases and 41% of benign lymphoepithelial lesions of the salivary glands.^11^, ^12^ Thus, even though it is not conclusive, it could be one more tool to help (but not define) the diagnosis. Unfortunately, in the current case, clonal detection in the paraffin-embedded uterine cervix sample was inconclusive.

Although a definitive diagnosis was not possible, the authors describe the association of two rare lymphoproliferative diseases and the diagnostic pitfalls of this group of diseases.

## Financial support

None declared.

## Authors' contributions

Jade Cury-Martins: Approval of the final version of the manuscript; design and planning of the study; drafting and editing of the manuscript; collection, analysis, and interpretation of data; effective participation in research orientation; critical review of the literature; critical review of the manuscript.

Marcelo Abrante Giannotti: Approval of the final version of the manuscript; collection, analysis, and interpretation of data; critical review of the manuscript.

Denis Miyashiro: Approval of the final version of the manuscript; collection, analysis, and interpretation of data; critical review of the manuscript.

Juliana Pereira: Approval of the final version of the manuscript; collection, analysis, and interpretation of data; critical review of the manuscript.

Jose Antonio Sanches: Approval of the final version of the manuscript; collection, analysis, and interpretation of data; effective participation in research orientation; critical review of the manuscript.

## Conflicts of interest

None declared.
