# Supplementary material for: A unique case of a lymphoproliferative disorder affecting the skin and uterine cervix on a male transgender
Source: An Bras Dermatol. 2023 Sep 1;99(1):135–9. doi: 10.1016/j.abd.2023.02.006 (PMC10964382; doi:10.1016/j.abd.2023.02.006)
Supplement: Supplementary file 1 [file mmc1.docx]

**ABD-D-22-00279_Supplementary** **material**

Methodology of the t(14;18) translocation investigation

**Supplementary material**

**Methodology of the t(14;18) translocation investigation**

The investigation of the t(14;18) translocation was performed using the methodology described by Kornacker et al., 2009 and the methodology described by Dongen et al., 2003.

For the methodology described by Kornacker et al., 2009, the following oligonucleotide pairs (Integrated DNA Technologies, USA) were used in two PCR steps (Nested PCR):

First step:

5’-GACCAGCAGATTCAAATCTATGGTGGT-3’

5’-GGACTCACCTGAGGAGACGGTGA-3’

Second step:

5’-CCTTTAGAGAGTTGCTTTACGTGGCC-3’

5’-GGAGACGGTGACCAGGGT-3’

The first step of PCR amplification was performed in a final volume of 25 µL containing 500 ng of genomic DNA, 10 µM of each oligonucleotide from the first step, 0.2 mM dNTP, 1.50 mM MgCl, 1x GoTaq Flexi Buffer, pH 8.3 and 1 U Taq DNA polymerase (Promega Corporation, USA). The conditions were: 1 cycle at 95°C for 2 min, 25 cycles at 95°C for 30s, 54°C for 40s, 72°C for 45s, 1 cycle at 72°C for 7 min and then 4°C∞, using an Applied Biosystems Veriti Thermal Cycler (Thermo Fisher Scientific, USA). The second step was performed in a final volume of 25 µL containing 5 µL of the PCR product obtained in the first step, 10 µM of each oligonucleotide from the second step, 0.2 mM each dNTP, 1.50 mM MgCl, 1x GoTaq Flexi Buffer, pH 8.3, and 1 U Taq DNA polymerase (Promega Corporation, USA). The conditions were: 1 cycle at 95°C for 2 min, 25 cycles at 95°C for 30s, 55°C for 40s, 72°C for 45s, 1 cycle at 72°C for 7 min and then 4 °C∞, using an Applied Biosystems Veriti Thermal Cycler (Thermo Fisher Scientific, USA).

For the methodology described by Dongen et al., 2003, the following sets of oligonucleotides were used in 3 different PCRs:

Tube A:

5’-GACCAGCAGATTCAAATCTATGG-3’

5’- ACTCTGTGGCATTATTGCATTATAT-3’

5’- CTTACCTGAGGAGACGGTGACC-3’

Tube B:

5’-GCACCTGCTGGATACAACACTG-3’

5’-AAACTAGCAGGGTGTGGTGGC-3’

5- GTAATGACTGGGGAGCAAATCTT-3’

5’- ACTGGTTGGCGTGGTTTAGAGA-3’

5’- CTTACCTGAGGAGACGGTGACC-3’

Tube C:

5’-CCTTCTGAAAGAAACGAAAGCA-3’

5’-TAGAGCAAGCGCCCAATAAATA-3’

5’-TGAATGCCATCTCAAATCCAA-3’

5’- CTTACCTGAGGAGACGGTGACC-3’

Each of the 3 PCRs were performed in a final volume of 25 µL containing 300 ng of genomic DNA, 10 µM of each oligonucleotide, 0.2 mM dNTP, 1.50 mM MgCl, 1x GoTaq Flexi Buffer, pH 8.3 and 1 U Taq DNA polymerase (Promega Corporation, USA). The conditions were: 1 cycle at 95°C for 2 min, 25 cycles at 95°C for 30s, 54°C for 40s, 72°C for 45s, 1 cycle at 72°C for 7 min and then 4°C∞, using an [Applied Biosystems Veriti Thermal Cycler](https://www.thermofisher.com/br/pt/home/life-science/pcr/thermal-cyclers-realtime-instruments/thermal-cyclers/veriti-thermal-cycler.html) ([Thermo Fisher Scientific](https://www.thermofisher.com/br/pt/home/life-science/pcr/thermal-cyclers-realtime-instruments/thermal-cyclers/veriti-thermal-cycler.html), USA).

After the PCRs described above, agarose gel electrophoresis was performed, containing GelRed^®^ Nucleic Acid Gel Stain dye (Biotium, USA) for later observation of the fragments under an UV transilluminator.

**References**

1. Kornacker M, Kornacker B, Schmitt C, Leo E, Ho AD, Hensel M. Commercial LightCycler-based quantitative real-time PCR compared to nested PCR for monitoring of Bcl-2/IgH rearrangement in patients with follicular lymphoma. Ann Hematol. 2009;88:43-50.

2. van Dongen J, Langerak A, Brüggemann M, Evans PAS, Hummel M, Lavender FL, et al. Design and standardization of PCR primers and protocols for detection of clonal immunoglobulin and T-cell receptor gene recombinations in suspect lymphoproliferations: Report of the BIOMED-2 Concerted Action BMH4-CT98-3936. Leukemia. 2003;17:2257–317.
